# The Chemical Characteristics and In Vitro Degradability of Pineapple By-Products as Potential Feed for Ruminants

**DOI:** 10.3390/ani13203238

**Published:** 2023-10-17

**Authors:** Dieu donné Kiatti, Alessandro Vastolo, Bossima Ivan Koura, Paola Vitaglione, Monica Isabella Cutrignelli, Serena Calabrò

**Affiliations:** 1 Department of Veterinary Medicine and Animal Production, University of Naples Federico II, 80138 Naples, Italy; alessandro.vastolo@unina.it (A.V.); monica.cutrigne@unina.it (M.I.C.); scalabro@unina.it (S.C.); 2 Ecole de Gestion et d’Exploitation de Système d’Elevage, Université Nationale d’Agriculture, Ketou P.O. Box 43, Benin; ivan.koura@una.bj; 3 Department of Agriculture, University of Naples Federico II, 80138 Naples, Italy; paola.vitaglione@unina.it

**Keywords:** fruit processing by-products, in vitro gas production, fiber, energy, volatile fatty acids, waste management, West Africa

## Abstract

**Simple Summary:**

Pineapple (*Ananas comosus* L.) fruit is mainly consumed fresh or after its transformation into juice, jam, candy, beverages, ice cream, powder, or wine. During processing, many leftovers are produced and released into the environment, contributing to pollution. This current study investigated the possible utilization of pineapple leftovers from two varieties (Smooth Cayenne and Sugarloaf) cultivated in West Africa. They were characterized individually (crown, bud end, peel, core, and pomace) regarding chemical composition, in vitro fermentation characteristics, and estimated metabolizable energy. As a result, the lipids and lignin content were at low levels and they would not negatively affect nutrient utilization in ruminants. However, the low dry matter and high sugar content in pineapples highlight preservation issues in their by-products. The core and pomace of pineapples showed low structural carbohydrate content, high in vitro degradability, and high volatile fatty acid production. This study suggests that pineapple by-products can be used in ruminant nutrition; the crown, bud end, and peel can be used as fiber sources, while core and pomace can be used as energy sources. In West Africa, these by-products could help farmers to supplement ruminants’ diet during dry seasons characterized by the scarcity of natural pasture which represent the main feedstuff for ruminant in this region.

**Abstract:**

Pineapple fruit, which is cultivated in tropical and subtropical areas, is processed by the food industry, generating a large amount of waste. Using pineapple by-products in animal nutrition could reduce feeding costs and contribute to the containment of pollution. The chemical composition and the in vitro fermentation of five pineapple by-products (crown, bud end, peel, core, and pomace) from two West African pineapple varieties (Smooth Cayenne—SC and Sugarloaf—SL) were evaluated. Significant differences were observed between the varieties and by-products. The dry matter (DM) content was low and superimposable between varieties, averaging 17.7%. On a DM basis, pomace showed the highest protein content (SC 8.10% and SL 8.81%, *p* < 0.001), whereas the crown showed the highest (*p* < 0.001) NDF content (47.62% and 39.01% for SC and SL, respectively). Due the high sugar content, the core and pomace showed high in vitro organic matter degradability (SC: 85.09% and SL: 83.98%), estimated metabolizable energy (SC: 7.91 KJ/kg and SL: 7.66 KJ/kg), and volatile fatty acid production (96.86 mmol/g and 90.62 mmol/g). Based on chemical composition and in vitro digestibility results, this study suggests that pineapple by-products have the potential to be used in ruminants’ diets, considering the crown, bud end, and peel as fiber sources and the core and pomace as substitutes or supplements to concentrate feedstuffs. Further research should be conducted on the storability of these by-products through in vivo trials evaluating animals’ performances and the quality of their products.

## 1. Introduction

The pineapple plant (*Ananas comosus* L.), belonging to the Bromeliaceae family, is originally from tropical and subtropical areas (Asia, South and North America, Africa, and Oceania), where the varieties mainly cultivated are Cayenne, Sugarloaf, Spanish, Queen, Pernambuco, and Perolera [1,2]. In 2021, worldwide pineapple production is estimated at 28,179,348 tons [3]. Costa Rica, Philippines, and Brazil are the major contributors. In West Africa, Nigeria and Benin are in the 8th and 17th positions, with 1,671,440 and 386,906 tons produced, respectively [4]. The high production of pineapple is due to its high demand at the global level and to the health-beneficial effect of pineapple fruit and pineapple-based products, such as juice, jam, candy, beverages, ice cream, powder, and wine. These products are known to be rich in many nutrients, such as vitamins (i.e., A, C, B_1_, B_6_, folic acid, beta-carotene), minerals (i.e., copper, manganese), and other compounds with antioxidant, digestive (bromelain), anti-inflammatory, and analgesic properties [5,6]. During fruit processing, a large quantity (about 65%) of leftovers is discarded as waste, including the peel (35.5%), core (14.7%), pomace (6.0%), bud end (4.6%), and crown (4.3%) [7,8]. Concerning the amount of fruit produced worldwide, more than 18 million tons of pineapple leftovers are generated annually. Generally, because of their short shelf life due to high moisture and sugar contents, these refusals are thrown away as waste material or, in a minor part, utilized for bromelain extraction or organic acid and ethanol production [5,9,10]. Their disposal in the environment causes serious problems such as air and underwater pollution. On the other hand, these leftovers may still contain traces of nutrients that can be useful as by-products for animal nutrition [11,12]. Particularly in West Africa, pineapple by-products could constitute a low-cost feed resource for utilization in the diets of grazing animals, especially as a supplement during the dry season. Therefore, utilizing pineapple by-products in animal nutrition could reduce breeders’ production costs and establish a circular economy by recycling the waste from factories that process the fruits.

Some studies investigated the use of pineapple by-products as feed for ruminants in different cultivation areas (i.e., Ethiopia and Southeast Asia) [13,14], evaluating the chemical composition and estimating the nutritional value. As suggested by some authors [15,16], dry or ensiled pineapple by-products could replace 50% of the roughage or corn silage in ruminants’ diets. In addition, little variation was noted between fresh and dry pineapple by-products in terms of chemical composition [7]. Some studies on pineapple waste [17,18] also investigated its sugar content (glucose, fructose, and sucrose), energy, and degradability. Other authors [19] focused on the in-situ degradability and proposed the inclusion level of pineapple cannery by-products in steer diets. In vitro degradable nitrogen was also investigated in pineapple waste [17,18]. Other authors experimented in vivo with pineapple by-products and concluded they could substitute roughage or concentrates in ruminants’ diets, improving dry matter intake, palatability, microbial activities, and animal performance [20,21,22,23].

However, pineapple by-products are still considered as waste because of their short shelf life for long-term utilization and their unknown nutritive values in other cultivation areas such as West Africa. In this region where the fruits are locally harvested and processed, pineapple by-products could be a useful resource in small ruminants production systems. This use would be helpful, particularly during the dry season, when the natural pasture becomes scarce [24,25]. However, before encouraging farmers to use them in animal feeding plans, it is necessary to know their individual nutritional characteristics considering influencing factors such as environmental conditions (i.e., temperature, rainfall, soil fertility), processing techniques, and plant varieties.

To fill this gap, this current study aimed to characterize, in terms of chemical composition, estimated metabolizable energy and in vitro fermentation characteristics of individual pineapple leftovers derived from the processing of two varieties (Sugarloaf and Smooth Cayenne) cultivated in the West African area.

## 2. Materials and Methods

### 2.1. Sampling Area

The pineapple (*Ananas comosus* L.) samples used in this study were cultivated in the Atlantic district of the Republic of Benin (6°18′–6°58′ N and 1°56′–2°30′ E); this area is in the sub-humid zone of West Africa. The Atlantic district is characterized by two rain seasons (March to July and mid-September to December) and two dry seasons (August to mid-September and December to March), with an annual average rainfall of 1200 mm, a temperature varying between 27 °C and 31 °C, and relative humidity between 69% and 97% [26].

### 2.2. By-Product Collection

Two varieties of pineapple (Sugarloaf and Smooth Cayenne) were directly collected from the field and transported to the zootechnic laboratory, Faculty of Agricultural Sciences (FSA), University of Abomey Calavi (UAC) for processing according to local use. The fruits were collected to represent the cultivation area; a total of 40 fruits from each variety were randomly collected from three farms in the Atlantic districts. First, the fruits were cleaned, and the crowns and bud ends were removed using a steel knife; successively, the peel and core were delicately separated from the pulp. Then, the pulps were pressed to remove the juice and retain the pomace using a hydraulic press machine (Tianyu Youdo Machine, model UDZL-W33, dimensions 330 × 330 × 600 mm, Kaifeng, China) used in small pineapple-processing units in West Africa. Finally, five by-products were generated for each variety from the fruits’ processing: the crown, bud end, peel, core, and pomace. Each obtained by-product was quantified, and the incidence (%) was estimated considering the weight of the whole fruit. About 300 g of each category of by-products was immediately dried in an oven at 65 °C until they reached a constant weight for dry matter evaluation [27]. In total, ten (10) samples (5 by-products × 2 varieties) were transferred to the laboratory of Feed Evaluation of the Department of Veterinary Medicine and Animal Production (University of Napoli Federico II, Napoli, Italy). Before the chemical analysis and in vitro trial, all the samples were grounded to pass through a 1 mm screen.

### 2.3. Chemical Composition

Chemical composition was carried out according to the official methods [27]; in particular, dry matter (DM), crude protein (CP), ether extract (EE), and ash contents were determined (934.01, 2001.11, 920.39, 942.05, for DM, CP, EE, and ash, respectively). Structural carbohydrates such as neutral detergent fiber (NDF), acid detergent fiber (ADF), and acid detergent lignin (ADL) were also assessed [28] with an Ankom 220 fiber analyzer (ANKOMTM Technology, Fairport, NY, USA). The filter bags used were burned to correct the NDF and ADF content.

### 2.4. Sugar Content

The quantitative determination of free sugars (FS) and total sugars (TS) was performed after mixing the by-products in distilled water at 1:40 and 1:80 ratios (w:w) for FS and TS, respectively. For the FS, the mixture was left at room temperature (20 °C) for 30 min, whereas for the TS, 20 mL of HCl (1:1) was added and then incubated in a thermostatic bath at 70 °C for 3 h; after cooling, three drops of 1% phenolphthalein were added. At this step, for both determinations, 5 mL of Carrez solutions (I and II) were added every 10 min followed by filtration. The obtained solution was titrated using Fehling’s solution (A and B) and 1% methylene blue [29]. The sugar content was calculated as (*f* × *d*)/(*a* − 0.1) where *f* is the factor due to the power of sugar (being 3.350 for reducing sugars and 5.150 for total sugars), *d* is the dilution factor, and *a* is the volume (ml) of the solution used during the titration.

### 2.5. In Vitro Fermentation

An in vitro gas production technique (IVGPT) was applied to evaluate the fermentation characteristics of pineapple by-products [30]. Each sample of pineapple by-products was weighed (1.0025 ± 0.0011 g) in four repetitions in 120 mL bottles with 75 mL of medium in anaerobic condition. Two bottles containing only the medium were used as blank. To study the in vitro fermentation profile, two reference feedstuffs (corn grain, *Zea mays* L. and Sulla hay, *Hedysarum coronarium* L.) were also used. Sheep rumen liquor (10 mL) containing its micro-flora flushed under anaerobic conditions was added to the bottles. The rumen liquor was collected in pre-warmed thermos from three adult ewes (Comisana × Sarda breed, 45–50 kg of live weight and 8–10 years old) slaughtered according to EU legislation (EC Council Regulation 882/2004). In the laboratory, it was pooled, mixed, and filtered through a double-thickness cheesecloth. The sheep were raised on pasture and were supplemented with 600 g/head/d of concentrate. All procedures involving animals were approved by the Ethical Animal Care and Use Committee of the University of Napoli Federico II (Prot. 2019/0013729 of 8 February 2019). The bottles were sealed with an aluminum cover and then incubated at 39 °C for 48 h. During the incubation period, the volume and the pressure of gas produced in the bottles were measured 22 times at 2–4 h intervals using a manual pressure transducer (Cole and Parmer Instrument Co., Vernon Hills, IL, USA). After each measurement, the bottles were shaken and replaced in the abovementioned conditions. The fermentation was stopped by cooling (4 °C), and pH was measured using a pH meter (model 720A+ Thermo Fisher Scientific, Rodano, MI, Italy). The supernatant liquors were sampled for the end-product analysis, and the remaining were filtered in pre-weight-sintered glass crucibles (Scott Duran, porosity #2). The crucibles were dried at 103 °C and burnt at 550 °C. Organic matter degradability at 48 h (OMD, %) was calculated as the difference between the incubated and residual OM.

### 2.6. End Product Analysis

Ten mL of supernatant liquor from each bottle was sampled at the end of incubation and centrifuged twice at 12,000× *g* for 10 min at 4 °C (Universal 32R centrifuge, Hettich FurnTech Division DIY, Melle-Neuenkirchen, Germany). Then, it was diluted (1:1) in 0.06 mol/l oxalic acid and injected into a gas chromatograph (Thermo-Quest 8000top Italia SpA, Rodano, Milan, Italy) equipped with a fused silica capillary column (30 × 0.25 mm, 0.25 μm film thickness, Supelco, Inc., Bellefonte, PA, USA) using pure acetic, propionic, butyric, iso-butyric, valeric, and iso-valeric acids as an external standard solution [31]. The total volatile fatty acid (tVFA, mmol/g), acetate/propionate ratio (A/P), and branched-chain fatty acids [BCFA = (iso-butyrate + iso-valerate)/tVFA × 100] were also calculated.

### 2.7. Processing Data

For each bottle, the cumulative gas volume was related to the incubated organic matter (OMCV, mL/g) and to the degraded organic matter (Yield, mL/g). The metabolizable energy (ME, KJ/kg DM) content in pineapple by-products was estimated using the following formula [32]:

ME=2.43+0.1206×G24+0.0069×CP+0.0187×EE

where CP, EE, and GP are, respectively, crude protein, ether extract, and the volume of gas produced by 200 mg of dry matter after 24 h of incubation with the rumen liquor.

Data of chemical composition, sugar content, estimated ME energy, and in vitro fermentation of pineapple by-products were statistically processed to verify the effect of varieties (Sugarloaf vs. Smooth Cayenne) and by-products (crown, bud end, peel, core, and pomace) according to the following model:

Y_ijk_ = µ + V_i_ + P_j +_ (V × P)_ij_ + ε_ijk_

where Y is the experimental data, µ represents the general mean, V the variety effect (i = 1,2), P is the effect of the by-product parts (j = 1, 2, 3, 4, 5), V × P is the interaction between the variety and by-products, and ε is the error term. GLM and Tukey’s HSD test were used in JMP software (Version 14 SW, SAS Institute Inc., Cary, NC, USA, 1989–2019). The distribution of data was checked by the Shapiro–Wilk test before any comparison, and the significant level was fixed at 5%.

## 3. Results

### 3.1. Pineapple By-Product Production

**Figure 1:**
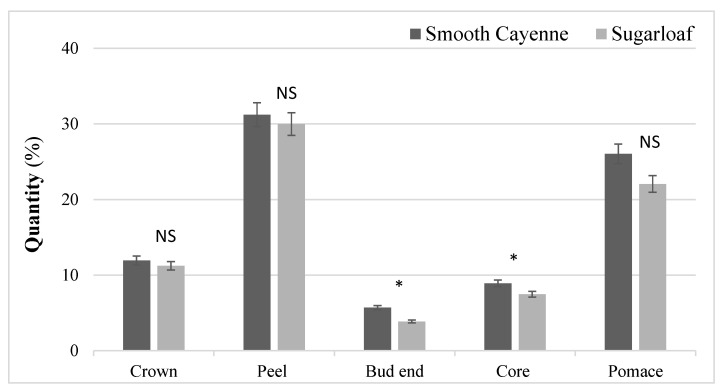
The amount of the two varieties of pineapple by-products obtained by processing the whole fruit. NS: non-significant between by-products, *: *p* < 0.05.

The percentage of pineapple by-products generated during the fruit processing are presented in Figure 1. The total by-products obtained during the processing represent 83.81% and 74.63% of whole fruit in Smooth Cayenne and Sugarloaf varieties, respectively. There were no differences between the varieties for the crown, peel, and pomace, averaging 11.59, 30.60, and 24.04%, respectively. However, the core (8.91 vs. 7.47%) and bud end (5.71 vs. 3.88%) were higher (*p* < 0.05) for the SC variety compared to SL.

### 3.2. Chemical Characteristics and Estimated Metabolizable Energy

Table 1 shows the chemical characteristics and estimated metabolizable energy value of the two pineapple by-product varieties. In general, high significant differences (*p* < 0.0001) were found between the two pineapples varieties (except ME, *p* = 0.0004), and between the by-products for all parameters, the interaction Variety × By-product was highly significant as well. The DM content ranged between 14.24% (core) to 20.38% (pomace) in Smooth Cayenne and between 15.08% (peel) to 20.98% (pomace) in Sugarloaf. The crown, peel, and pomace showed significantly higher CP content (*p* < 0.0001) than the bud end and core in both varieties. Ash content was high in the crown (6.08 vs. 4.81% DM), bud end (6.22 vs. 4.59% DM), and peel (5.62 vs. 4.93% DM) compared to the core and pomace (2.48 vs. 1.58 and 3.00 vs. 2.78% DM) in SC and SL, respectively, even if in Smooth Cayenne, the values were always higher. In both pineapple varieties, the highest (*p* < 0.001) EE and structural carbohydrate (NDF and ADF) values were found in the crown and the lowest in the core. Regarding ADL, the highest values were found in the Smooth Cayenne crown (3.09% DM; *p* < 0.0001) and in the Sugarloaf bud end (2.19% DM) and crown (2.08% DM), and the lowest values were found in the pomace of both varieties.

Regarding sugars, the Smooth Cayenne crown showed the lowest content of TS and FS, whereas the core showed the highest (*p* < 0.0001) TS, and the pomace showed the highest FS content. On the other hand, for Sugarloaf, the pomace showed the lowest content of TS whereas the crown the lowest content of FS; the core showed the highest levels for both parameters. 

The estimated ME content was significantly (*p* < 0.001) higher in the core and pomace and lower in the crown and bud end, while the estimated ME of the peel was intermediate, even if it was not significantly different from the core and pomace in the SL variety.

### 3.3. In Vitro Degradability, Gas Production, and VFA Profiles

The in vitro fermentation characteristics of the two varieties of pineapple by-products are reported in Table 2. The by-products significantly affected all parameters (*p* < 0.0001), even if the volume of gas related to the degraded organic matter (Yield) was significantly affected by both factors (variety and by-products).

The pH was significantly (*p* < 0.0001) different in the by-products of each variety; in any case, it ranged from 5.92 to 6.46, which indicates a correct fermentation process [33]. After 48 h of incubation, organic matter degradability (OMD) ranged from 56.14% (in the crown) to 85.35% (in the pomace) in the Smooth Cayenne variety and from 62.61% (in the crown) to 86.55% (in the core) in the Sugarloaf variety. Large variations were also found in the cumulative volume of gas between the pineapple varieties and among the five by-products within the variety. In particular, OMCV varied from 146.51 to 244.72 mL/g for Smooth Cayenne and from 158.52 to 255.02 mL/g for Sugarloaf. Regarding the Yield parameter, a high variability was also found in the Smooth Cayenne variety (from 242.07 to 314.25 mL/g), but a low variability was found in Sugarloaf (from 253.21 to 304.62 mL/g). The core and pomace showed the lowest pH values with the highest OMD, OMCV, and Yield (*p* < 0.001) in Smooth Cayenne by-products; the same result was observed in Sugarloaf by-products, except for Yield.

**Figure 2:**
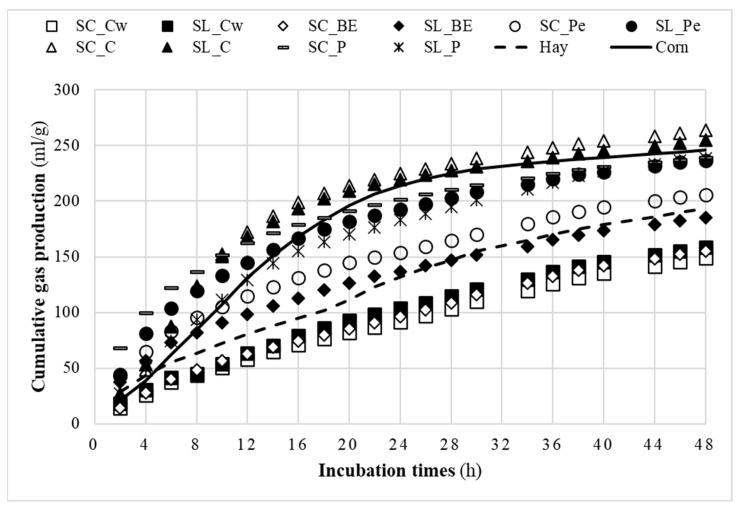
In vitro fermentation profile in the two varieties of pineapple by-products along 48 h of incubation. SL: Smooth Cayenne, SL: Sugarloaf, Cw: Crown, BE: Bud end, Pe: Peel, C: Core, and P: Pomace.

The in vitro fermentation profile in pineapple by-products within 48 h of incubation is pictured in Figure 2. The curve of corn grain and Sulla hay, incubated as reference feedstuffs, were also represented. Three groups characterized by low, medium, and high fermentation can be distinguished. The least fermentable, lower than Sulla hay, were the crowns of both varieties and the SC bud end, while the most fermentable were the cores and pomaces for both varieties and the SL peel, which were similar to corn grain. In the middle, the SC peel and SL bud end with a fermentation profile superimposable on Sulla hay.

Table 3 shows the in vitro fermentation-end products in the two varieties of pineapple by-products obtained after 48 h of incubation. All factors, showed a significant effect (*p* < 0.005), even if the same trend was not always observed in both varieties, as demonstrated by the statistical significance of the interaction values presented in the table. 

In Smooth Cayenne, the acetate and acetate/propionate ratio were the highest in the crown, whereas propionate and butyrate were the highest in the core and pomace, respectively. Regarding Sugarloaf, the highest (*p* < 0.001) amount of acetate, propionate, and butyrate was found in the core, while the acetate/propionate ratio was the highest (*p* < 0.0001) in the crown. The highest (*p* < 0.0001) amount of valeric acid was observed in the crown and core of Smooth Cayenne and in the core and pomace of Sugarloaf, while the lowest was obtained in the bud end for both varieties. For Smooth Cayenne and Sugarloaf varieties, the percentage of BCFA was significantly (*p* < 0.001) higher in the crown and lower in the core. However, the core always showed the highest (*p* < 0.0001) total VFA production.

The correlation between chemical composition and the IVGPT parameters of pineapple by-products is presented in Table 4. The results showed that the OMD, OMCV, and Yield were significantly and negatively correlated with EE, Ash, NDF, and ADF, while pH was positively correlated with the same parameters.

In addition, OMD and OMCV were positively (*p* < 0.001) correlated with ME and negatively (*p* < 0.01) with ADL. Regarding sugar, FS was correlated with OMD (*p* < 0.05), and TS was correlated with pH and tVFA (*p* < 0.05 and *p* < 0.01, respectively). Any fermentation parameters were correlated with CP content.

## 4. Discussion

In this study, the individual by-products of two varieties of pineapple (Sugarloaf and Smooth Cayenne) cultivated in West Africa were characterized in terms of chemical composition, estimated ME, and in vitro fermentation parameters for their possible use in ruminant nutrition. The hypothesis is that some differences exist between the by-products of the fruits obtained during the processing.

### 4.1. Chemical Characteristics and Estimated ME Energy: Comparing By-Products and Variety

In our study, the amounts of pineapple by-products obtained after fruit processing nearly double those (skin, shoot tip, core, stems, crowns) reported by other investigations [8,14], except for the peel. In terms of chemical composition, in other studies on pineapple by-products [8,13,14,19,23], it is reported that CP, EE, NDF, and ADL range from 4.4–5.7, 0.2–1.2, 20.5–44.69, and 23.04–39.40% DM, respectively. The same references also quantified organic matter (OM), non-fiber carbohydrate (NSC), and water-soluble carbohydrate (WSC), resulting, respectively, in 95.54, 48.8, and 5.4% DM. In our investigation, a low DM content was found in all by-products (14.24–20.98%). Ash, ether extract, and structural carbohydrates were present in decreasing quantities from the crown, bud end, peel, pomace to core in both varieties. The EE and ADL were low in all pineapple by-products for both varieties (0.20–1.06, 1.27–3.09% DM). Regarding the categories, the crown and bud end pineapple by-product showed the highest lignin content compared to the peel, core, and pomace, whereas a low variation in crude protein was found between by-products. As expected, the range observed in the chemical composition is due to the parts of the by-products, which are different in terms of texture. The processing technique also influences the difference in terms of chemical composition between the by-products generated. The range of variation in the chemical composition found in this current study is in line with other research on several kinds of agro-industrial by-products [34,35]. An average fat content of 3.08% has been reported in previous studies [36,37,38]. This is 0.43% higher than the fat determined in this study. However, compared with the cited studies, the ADL content was similar. On the other hand, the structural carbohydrate (NDF and ADF) of the crown, bud end, and peel reported in this study for both varieties were comparable to the results already published on the same by-products [19,39]. The NDF and ADF of the core and pomace were low and not much different from other by-products such as sugar beet pulp, pepper core, and citrus pulp [35]. However, some studies highlight the high proportion of NDF and ADF in dehydrated pineapple by-products [38,40], comparable to the grass grown in West African natural pasture [30]. This discrepancy might be due to the method of collection (separately vs. pooled: bud end, peel, core, and pomace together), processing techniques (homemaking vs. industrial), and cultivation area conditions (soils, temperature, and rainfall differences) [41].

In this study, for both varieties, the crude protein level was higher in the crown, peel, and pomace (7.51–8.81% DM) compared to the bud end and core (5.18–6.54% DM). The crude protein content of pooled pineapple by-products (peel, bud end, core, and pomace) investigated in South America was higher than those obtained individually in the bud end and core but similar to the CP of the crown, peel, and pomace [40,42,43]. The crude protein reported in these papers is low (for the bud end and core) and similar (for the crown, peel, and pomace) compared to the range of 7–8% recommended for the efficient functioning of rumen microorganisms [28]. That means that there is the necessity of introducing protein source ingredients in the diet for ruminants when pineapple by-products are used.

Regarding the estimated metabolizable energy content, the highest values were obtained in the core and pomace, which also showed the highest sugar content (TS and FS) for the Smooth Cayenne and Sugarloaf varieties. As revealed previously, the feedstuffs which are rich in sugar as some pineapple by-products (e.g., core, pomace) could be useful as an energy source in ruminant nutrition [17,44]. Our findings were in accordance with previous research on pineapple by-products in terms of sugar content [14,45]. They observed that pineapple by-products contain high levels of sugar (40–75%), mainly constituted by sucrose (70%) and then by glucose (20%) and fructose (10%), which are primary energy sources when they are present in animals’ diet. The ME that we estimated was close to the results obtained by [40] in Brazil and fall into the interval (10.7–14.5 MJ/kg DM) observed by [13] in the classification of fruit by-products rich in energy. However, some authors [5,37] have observed the lowest sugar content (26.28%) in pineapple by-products compared to our data. These results suggested separating the pineapple by-products into two groups, ME and CP sources, before their inclusion in ruminants’ diet.

### 4.2. In Vitro Fermentation Characteristics: Comparing By-Products and Varieties

Regarding the in vitro fermentation characteristics, data showed that the IVGPT is a suitable method to study pineapple by-products when incubated for 48 h with sheep rumen fluid. In fact, the pH values (ranging from 5.92 to 6.46) varied within the normal interval rumen pH value. Moreover, as reported in previous studies [46], significant correlations were observed underline the accordance between chemical composition and in vitro parameters: the increase in structural carbohydrates, ether extract, and ash reduced gas, VFA production, and OM degradability, whereas the increase in sugar and energy increased these parameters.

The results that emerged from in vitro fermentation show that the by-products with the highest amount of sugar and energy (core and pomace for both varieties) also had the highest organic matter degradability, cumulative gas production related to incubated OM (OMCV), and cumulative gas production related to degraded organic matter (Yield). These findings support the fact that microorganisms utilized energy from by-products during the fermentation for their own growth, converting the structural carbohydrate to end products (i.e., volatile fatty acid, carbon dioxide, and methane) [47]. Regarding the end products, the acetate/propionate rations are higher for the crown, bud end, and peel in both varieties (Ace/Pro > 1.0), while their total volatile fatty acid production is lower compared to the core and pomace. Regarding OM degradability, our results for the crown and bud end are consistent with those reported in the literature [18]. The higher OMD observed for core and pomace as well as for peel in this study are comparable to data published on in vitro trials carried out incubating pineapple waste with cow rumen fluid [13,14].

The volatile fatty acid produced by microorganisms in the rumen represent the energy source for ruminants. High-level of volatile fatty acid means high energy available to satisfy requirements for maintenance and production (meat or milk). The total VFA recorded in this study was revealed to be higher than those reported in other investigations on pineapple by-products [20,36], as well as on other by-products such as banana peels, papaya peels, mango seed kernels, and tomato pomaces [13,48]. Several factors such as sampling and chemical composition might affect volatile fatty acid production, which affects pH and microorganism development in the rumen. Surprisingly, previous research focused on natural pasture and cultivated grass in the pineapple growing area of West Africa and reported similar results regarding the total VFA [30]. These results might encourage further research to replace limited feedstuffs during the dry season with alternative resources, such as pineapple by-products, to help the farmers to sustain their productivity through the dry season and maintain animal productivity throughout the year.

To better discuss the fermentation profile of the different pineapple by-products, the in vitro cumulative gas production recorded during the 48 h period of incubation is represented in Figure 2, together with the corn grain and Sulla hay incubated as reference feedstuffs for energy and fiber sources, respectively. Three different groups of feedstuffs, in terms of fermentation patterns, are evidenced. The first group is composed of the crowns of both varieties and the bud end of the Smooth Cayenne variety, characterized by slow and low fermentability and more comparable to a forage of less quality. The second group is composed of pineapple by-products, which had fermentation characteristics similar to Sulla hay and concern the Sugarloaf bud end and Smooth Cayenne peel. The third group contains by-products (Smooth Cayenne: pomace and core; Sugarloaf: peel, pomace, and core) similar to corn grain, with a high fermentation rate in the first hours of incubation. However, the main differences are clearer in the first six hours of incubation for the first group, whereas the differences are clearer around 14 h and 48 h for the second and the third group, respectively. Moreover, the profile of the first and second groups of by-products was quite linear, meaning that an asymptote had not been achieved at this time of incubation (48 h) due to the high NDF content. Possibly, in order to better observe the characteristics of fermentation, more hours of incubation would have been necessary for this group of pineapple by-products.

### 4.3. Problems and Opportunities in Ruminant Diets

The low DM content and high sugar level found in all by-products (as reported before) suggest a risk of rapid decomposition after the processing. This finding notes the long-term conservation problem and the difficulty of using them in animal feeding plans. Pineapple by-products, as fruit waste in general, were already noted to be problematic in terms of preservation [7]. However, the presence of easily fermentable sugars (free sugars) in these by-products justifies the availability of energy that would be useful by the microorganism in the rumen and, as reported [21,22,49], could increase dry matter intake, palatability, and animal performance by improving microbial activity and nitrogen utilization. Consequently, high microbial activity would contribute to enhance the amount of digestible protein in the gut via microbial protein contribution.

The low content of EE (>5% per kg DM) and ADL in the by-products of both varieties is interesting because when these parameters are high in a ruminants’ diet can depreciates the diet by affecting nutrient intake and digestibility. Consequently, the utilization of pineapple by-products as ingredients could reduce the amount of EE and ADL in the final diet, making it efficient for ruminants. A decrease in nutrient intake (DM, OM, CP, and NDF) was observed with an increase in EE up to 7.9% [50], while the DM and OM degradability of grape seed, dried tomato pulp, and pepper core by-products were low because of their high level of ADL [35].

Regarding the CP content, it was lower than the minimum concentration (7% of total DM) for a normal functionality of the rumen in some by-products (bud end and core). The lowest CP content (3.5% DM) was reported when pineapple waste was ensiled with cassava root meal or cassava peel (4:1 ratio) for preservation issues [38]. These by-products cannot be utilized alone as protein sources in ruminant diets. On the other hand, interestingly, they showed energy content similar to the natural pastures of West Africa, which become scarce during the dry season [30]. Pineapple by-products could be useful in this case to replace the grass (e.g., Poaceae Fam.) during the dry season, a critical period for grassing animals in the West African region. However, as mentioned above, the maturity status of pineapples, the processing technology applied, and environmental parameters can fluctuate the amount of sugar and ME in these by-products.

In addition, the utilization of pineapple peels up to 20% in ruminants’ diets formulated with 10% milled corn and 70% bran was revealed to improve feed intake and organic matter digestibility. The ability of pineapple by-products to enhance the intake and digestibility might be due to their sweetness and the presence of the bromelain enzyme, which is known to improve digestibility [19,36]. Some authors also reported an improvement in the quality of animal products when pineapple by-products were used in the ruminants’ diet as feed ingredients [51,52]. In 2022, Bulkaini reported that the addition of pineapple by-products fermented with lactic acid bacteria at levels of 20% can improve the production performance and carcass quality of male Bali cattle.

Based on this aspect, pineapple by-products can substitute a part of conventional feedstuffs in West African ruminants’ diets as already reported for other fruit waste [53,54].

## 5. Conclusions

Our results suggested that pineapple by-products have the potential to be used as feed ingredients in ruminants’ feeding plans according to their chemical composition and in vitro fermentation parameters if they are properly and separately collected during fruit processing. The results suggested considering the crown, bud end, and peel as fiber sources (e.g., roughage), while the core and pomace could be substitutes for the concentrate feedstuffs in ruminants’ diets. However, due to the high amount of sugar still present in pineapple by-products, their use at high levels in a diet could cause digestive disturbances. The results evidence the possibility of using pineapple by-products in animal nutrition; however, further studies must be conducted to determine the way it would be preserved for a long time. Additional studies are necessary to determine the right amount of pineapple by-products to include in West African ruminants’ diets without affecting animal health as well as the performance, welfare, and quality of products. Further research must focus on some points: ensiling pineapple by-products with hay to enhance self-life and testing the in vivo effect of pineapple by-products to evaluate the performance, health status, and quality of the products (meat and milk). It is also important to standardize the techniques in collecting and processing pineapple fruits. Moreover, further data relating to by-product transport costs in terms of money and carbon emissions would be helpful to demonstrate the contribution to the circular economy that the use of these feedstuffs can make.

## Figures and Tables

**Table 1:** Chemical characteristics and estimated energy value of the two varieties of pineapple by-products.

Items	DM	CP	Ash	EE	NDF	ADF	ADL	TS	FS	ME
	%	%DM						%	%TS	MJ/kg DM
Smooth Cayenne variety										
Crown	17.63B	7.55A	6.08A	1.06A	47.62A	26.03A	3.09A	41.20D	26.28D	5.16C
Bud end	15.79C	5.41B	6.22A	0.48B	45.60B	21.28B	2.34B	57.23B	28.92D	5.11C
Peel	15.63C	7.51A	5.62B	0.45B	33.85C	16.61C	1.77C	52.15C	35.40C	6.30B
Core	14.24C	6.43B	2.48D	0.20C	13.46E	7.05E	1.83BC	70.43A	43.70B	7.82A
Pomace	20.38A	8.10A	3.00C	0.24C	19.58D	9.40D	1.55C	68.10A	62.04A	7.99A
Sugarloaf variety										
Crown	20.62A	8.48A	4.81B	0.68A	39.01A	20.31A	2.08AB	54.94C	24.63B	5.62B
Bud end	17.07B	6.54B	4.59B	0.37B	33.27B	15.19B	2.19A	58.87B	36.91A	5.94B
Peel	15.08C	8.01A	4.93A	0.35B	26.48C	11.97C	1.60B	58.44B	27.79B	7.34A
Core	15.29C	5.18B	1.58D	0.19C	10.80E	6.85E	1.59BC	84.96A	40.40A	7.95A
Pomace	20.98A	8.81A	2.78C	0.30BC	21.53D	9.75D	1.27C	40.81D	36.58A	7.36A
*p*-value										
Variety	<0.0001	<0.0001	<0.0001	<0.0001	<0.0001	<0.0001	<0.0001	<0.0001	<0.0001	0.0004
By-product	<0.0001	<0.0001	<0.0001	<0.0001	<0.0001	<0.0001	<0.0001	<0.0001	<0.0001	<0.0001
Variety × By-product	0.0004	<0.0001	<0.0001	<0.0001	<0.0001	<0.0001	<0.0001	<0.0001	<0.0001	<0.0001
MSE	0.9033	0.2758	0.0053	0.0004	0.1440	0.0423	0.0171	0.8630	0.9850	0.0777

DM: dry matter, CP: crude protein, EE: ether extract, NDF: neutral detergent fiber, ADF: acid detergent fiber, ADL: acid detergent lignin, TS: total sugar, FS: free sugar, ME: metabolizable energy. Along the column, for each variety, different letters indicate the statistical difference (*p* < 0.001). MSE: mean square error.

**Table 2:** In vitro fermentation characteristics in the two varieties of pineapple by-products.

Items	pH	OMD	OMCV	Yield
		%	ml/g	ml/g
	Smooth Cayenne variety			
Crown	6.46A	56.14D	146.51C	259.45C
Bud end	6.34B	62.83C	154.98C	242.07D
Peel	6.31B	75.95B	205.91B	271.72B
Core	5.98D	84.82A	244.72A	314.25A
Pomace	6.19C	85.35A	238.75A	285.26B
	Sugarloaf variety			
Crown	6.44A	62.61D	158.52C	253.21D
Bud end	6.40A	65.17D	182.60B	281.96C
Peel	6.34B	75.91C	236.71A	304.62A
Core	5.92D	86.55A	255.02A	284.87BC
Pomace	6.18C	81.41B	240.03A	296.35AB
*p*-value				
Variety	0.8941	0.0135	<0.0001	<0.0001
By-product	<0.0001	<0.0001	<0.0001	<0.0001
Variety × By-product	<0.0054	<0.0001	0.0141	<0.0001
MSE	0.0010	2.5070	45.2111	15.1512

OMD: organic matter degradability (% of incubated OM), OMCV: cumulative volume of gas related to incubated OM, Yield: cumulative volume of gas related to degraded OM. Along the column for each variety, different letters indicate the statistical difference (*p* < 0.001). MSE: mean square error.

**Table 3:** In vitro fermentation end-products in the two varieties of pineapple by-products.

Items	Ace	Pro	Iso-But	But	Iso-Val	Val	tVFA	BCFA	Ace/Pro
	mmol/g							%	
	Smooth Cayenne variety								
Crown	33.51A	25.44C	0.46A	19.36C	0.66A	0.67A	80.10C	1.61A	1.51A
Bud end	29.29B	24.22C	0.31B	21.26C	0.39B	0.43B	75.90C	1.06B	1.38B
Peel	25.20C	23.05C	0.38B	24.88B	0.46B	0.45B	74.43C	1.30B	1.24C
Core	27.04BC	50.05A	0.17C	25.26B	0.22C	0.70A	103.44A	0.43C	0.62E
Pomace	19.96D	31.77B	0.38B	37.02A	0.62A	0.53B	90.28B	1.37A	0.78D
	Sugarloaf variety								
Crown	25.59BC	21.05D	0.38A	17.15C	0.59A	0.48C	65.25C	1.67A	1.36A
Bud end	24.50C	22.57CD	0.33AB	23.63B	0.48AB	0.39C	71.92B	1.26B	1.22B
Peel	27.08B	24.23C	0.26BC	24.24B	0.46B	0.66B	77.93B	1.05BC	1.23B
Core	30.24A	50.79A	0.17D	27.17A	0.22C	0.92A	109.53A	0.39D	0.66C
Pomace	21.89D	36.09B	0.21C	12.24D	0.25C	1.02A	71.70B	0.77C	0.72C
*p*-value									
Variety	0.0010	<0.0001	<0.0001	<0.0001	0.0002	<0.0001	<0.0001	0.0034	<0.0001
By-product	<0.0001	<0.0001	<0.0001	<0.0001	<0.0001	<0.0001	<0.0001	<0.0001	<0.0001
Variety × by-product	<0.0001	0.0004	<0.0001	<0.0001	<0.0001	<0.0001	<0.0001	<0.0001	0.0001
MSE	0.9510	1.4491	0.0008	0.7540	0.0021	0.0020	6.0990	1.3470	0.0020

Ace: acetate, Pro: propionate, Iso-But: iso-butyrate, But: Butyrate, Iso-Val: iso-valerate, Val: valerate, tVFA: total volatile fatty acid, BCFA: branched chain fatty acid, Ace/Pro: acetate to propionate ratio. Along the column for each variety, different letters indicate the statistical difference (*p* < 0.001). MSE: mean square error.

**Table 4:** Correlation between chemical composition and the in vitro fermentation characteristics of pineapple by-products (n = 20).

	pH	OMD	OMCV	Yield	tVFA
**CP**	0.40	−0.03	0.01	0.14	−0.56
	NS	NS	NS	NS	NS
**EE**	0.75	−0.85	−0.83	−0.65	−0.48
	*	**	**	*	NS
**Ash**	0.87	−0.84	−0.83	−0.67	−0.70
	**	**	**	*	*
**NDF**	0.90	−0.95	−0.95	−0.81	−0.72
	***	***	***	**	*
**ADF**	0.86	−0.95	−0.96	−0.83	−0.63
	**	***	***	**	NS
**ADL**	0.61	−0.85	−0.85	−0.63	−0.22
	NS	**	**	NS	NS
**FS**	−0.56	0.70	0.59	0.41	0.53
	NS	*	NS	NS	NS
**TS**	−0.72	0.59	0.54	0.31	0.80
	*	NS	NS	NS	**
**ME**	−0.83	0.96	0.98	0.83	0.67
	**	***	***	**	*

CP: crude protein, EE: ether extract, NDF: neutral detergent fiber, ADF: acid detergent fiber, ADL: acid detergent lignin, FS: free sugar, TS: total sugar, ME: metabolizable energy. OMD: organic matter degradability, OMCV: cumulative volume of gas related to incubated OM, Yield: cumulative volume of gas related to degraded OM, tVFA: total volatile fatty acid. NS: non-significant, *, **, ***: indicate *p* < 0.05, *p* < 0.01, *p* < 0.001, respectively.

## Data Availability

The data of this study are available from the corresponding author.
